# Relationship of Seminal Oxidation-Reduction Potential with Sperm DNA Integrity and pH in Idiopathic Infertile Patients

**DOI:** 10.3390/biology9090262

**Published:** 2020-09-01

**Authors:** Sergio Garcia-Segura, Jordi Ribas-Maynou, Sandra Lara-Cerrillo, Agustín Garcia-Peiró, Ana Belén Castel, Jordi Benet, Maria Oliver-Bonet

**Affiliations:** 1 Unit of Cell Biology and Medical Genetics, Department of Cell Biology, Physiology and Immunology, Universitat Autònoma de Barcelona, Av. Can Domenech s/n, Bellaterra, 08193 Catalunya, Spain; j.ribas87@gmail.com; 2 CIMAB, Barcelona Male Infertility Centre, C. Vallcorba 1-3, Sant Quirze del Vallès, 08192 Catalunya, Spain; info@cimab.es (S.L.-C.); agusti.garcia@uab.cat (A.G.-P.); 3 Instituto de Fertilidad, C. Calçat 6, 07011 Palma Mallorca, Spain; abcastel@yahoo.es; 4 Genosalut, Camí dels Reis 308, 07010 Palma Mallorca, Spain

**Keywords:** sperm, oxidative stress, oxidation-reduction potential, sperm DNA fragmentation, DNA damage, comet assay, DNA compaction, chromomycin A3, pH, male infertility

## Abstract

Seminal oxidative stress (OS) is one of the most promising factors to describe the causes of idiopathic male infertility. Redox balance is essential in several biological processes related to fertility, so alterations such as high reactive oxygen species (ROS) levels or low antioxidant agent levels can compromise it. MiOXSYS has been developed to evaluate the seminal static oxidation-reduction potential (sORP) and it has been proposed as an effective diagnostic biomarker. However, its relationship with parameters like sperm DNA fragmentation (SDF), chromatin compaction status or seminal pH requires further analysis, making it the object of this study. Semen and sORP analysis were performed for all samples. A terminal deoxynucleotidyl transferase dUTP nick end labeling assay (TUNEL) and Comet assay were used to assess SDF and chromomycin a3 (CMA3) test to assess sperm chromatin compaction. Regarding sORP measures, it was found that alkaline pH has an effect on sample reproducibility. To our knowledge, this unexpected effect has not been previously described. A statistical analysis showed that sORP correlated negatively with CMA3 positive cells and sperm motility, but not with SDF. As redox dysregulation, which occurs mainly at the testicular and epididymal level, causes chromatin compaction problems and leaves DNA exposed to damage, an excess of ROS could be counterbalanced further by a seminal supply of antioxidant molecules, explaining the negative correlation with CMA3 positive cells but no correlation with SDF. Our results show that the study of idiopathic infertility would benefit from a combined approach comprising OS analysis, SDF and chromatin compaction analysis.

## 1. Introduction

Male infertility is a multifactorial disorder that is present in approximately half of the population of infertile couples [1,2,3,4]. Several authors have pointed out that a standard semen analysis, including sperm motility, concentration, morphology and physiological semen parameters, has limited diagnostic value [5,6,7]. Indeed, about 15% of infertile men present normozoospermia and idiopathic causes, and so require an in-depth study to find a more accurate diagnosis [2,8]. Together, these facts highlight the need to improve male infertility diagnoses by adding relevant biomarkers, which will bring to light new biological mechanisms that allow a better understanding of infertility etiology.

A correct redox balance is essential for activating processes such as sperm capacitation, sperm hyperactivation, acrosomal reaction or oocyte fusion [9,10,11,12,13]. Over recent decades, a number of studies have shown that redox deregulation, either by an increase in reactive oxygen species (ROS) or a decrease in antioxidant activity, results in alterations in sperm motility, lipid peroxidation or sperm DNA fragmentation (SDF) [13,14,15,16]. Additionally, several works have described that infertile men have higher concentrations of ROS and lower levels of antioxidants [17,18,19,20]. Oxidative stress (OS) assessments are usually based on ROS level measurements in seminal plasma, but antioxidant levels, which can offset or exacerbate this cellular stress, are not usually calculated. A new methodology based on an electrochemical analysis of the oxidation-reduction potential (ORP), the MiOXSYS system, has recently been developed [21]. This system analyzes the seminal redox balance and has been proposed as an effective tool for the diagnosis of male infertility [22]. However, it is described that devices’ measurements may be affected by seminal viscosity [23,24], but it is unknown whether it may be affected by other characteristics of seminal plasma.

Sperm DNA fragmentation (SDF) has also been established in recent decades as a biomarker for male infertility diagnoses. Different studies have shown that SDF values are inversely related to sperm motility and other reproductive health indicators, such as fertilization rates, implantation rates, or recurrent miscarriages [25,26,27,28,29,30,31], especially for in vitro fertilization. Terminal deoxynucleotidyl transferase dUTP nick end labeling (TUNEL) and Comet assays display a very accurate sensitivity and specificity for predicting fertility rates [32,33]. The TUNEL assay is based on the detection of fluorescent labeling of the 3′ free ends resulting from DNA breaks. The Comet assay is based on the release of nuclear protamines from the sperm chromatin and subsequent single-cell electrophoresis that allows the migration of DNA fragments to the positive pole, generating a comet-like tail. Additionally, the Comet assay is the only method that allows the quantitative measurement of DNA damage individually for each cell and the only one that detects the incidence of single-strand and double-strand breaks separately, depending on alkaline or neutral pH conditions, respectively [29]. Previous studies have demonstrated a direct correlation between seminal ROS and single-strand SDF [10,33,34].

Sperm chromatin condensation is also a parameter that has been effective in explaining male infertility. Several studies show that deficiencies in protamination have been associated with sperm head morphological defects, SDF, chromosome reorganization and varicocele patients [35,36,37,38,39]. The relationship between chromatin condensation, oxidative stress and DNA damage has been extensively studied [35,40,41]. As a result, comprehensive models like the double-step hypothesis suggested by Aitken and de Iuliis have been proposed, stating that defects in chromatin remodeling can leave exposed areas that can subsequently be damaged by oxygen radicals, thus increasing the total amount of sperm DNA fragmentation [42].

The aim of the present study is to evaluate the relevance of ORP in idiopathic male infertility. To do so, the relationship between ORP and sperm DNA integrity biomarkers, such as DNA damage and chromatin condensation, has been analyzed. Additionally, the possible influence of seminal parameters, such as pH, on static oxidation-reduction potential (sORP) measurement has been evaluated.

## 2. Materials and Methods

### 2.1. Sample Collection

Seminal samples were obtained from male idiopathic patients from Instituto de Fertilidad of Palma de Mallorca, in collaboration with Genosalut and Universitat Autònoma de Barcelona. A total of 42 samples were collected, with 2–5 days of sexual abstinence. Samples were frozen immediately after liquefaction and transported to the Universitat Autònoma de Barcelona (UAB), where they were stored in liquid nitrogen at −196 °C until its analysis. Prior to cryopreservation, a basic semen analysis (sperm concentration, motility, morphology and pH) was performed with the Sperm Class Analyzer (SCA, Microptic, Barcelona, Spain), Sperm Blue (Microptic) and pH-indicator strips, according to the 2010 WHO guidelines [43]. Seminal viscosity was measured using 20 micron Leja chambers as previously suggested Rijnders [44], showing the results in centipoise (cps). All donors signed their informed consent and the study was approved by the Ethics Committee of Corporació Sanitaria Parc Taulí (Ref.: 2014676).

### 2.2. Measurement of Oxidation Reduction Potential

The ORP was measured using the MiOXSYS system (Aytu BioScience, Englewood, CO, USA). As instructed in the manufacturer’s protocol, 30 µL of each sample were placed in the sensor [21,23]. Samples were analyzed twice consecutively in the same conditions. The electronic device reads the electron flux voltage from seminal redox reactions with a galvanostatic-based sensor and the displayed result is the static ORP (sORP) in millivolts (mV). The average of the two values obtained was subsequently normalized by the sperm concentration (nsORP-C) (mV/10^6^ sperm/mL), seminal viscosity (nsORP-V) (mV/cps) and pH (nsORP-P) (mV/U pH). The cut-off value was set at 1.34 mV/10^6^ sperm/mL to discern between good quality seminal samples (≤1.34 mV/10^6^ sperm/mL) from altered seminal samples (>1.34 mV/10^6^ sperm/mL), as has been established before [45]. Five samples were excluded after the device failed to measure their sORP, due to their high viscosity.

### 2.3. Sperm DNA Fragmentation Analysis

All samples were evaluated using TUNEL and Comet assays. An In Situ Cell Death Detection Kit (Ref. 11684795910, Roche Diagnostic GmbH, Penzberg, Germany) was used for the TUNEL assay as previously described [46]. The semen samples were washed three times in phosphate buffered saline solution (PBS) before adjusting the sperm concentration to 5 × 10^6^ cells/mL. Resuspended sperm samples were divided into three 200 µL tubes, fixed with 4% paraformaldehyde for 1 h at room temperature and then washed in PBS supplemented with 1% bovine serum albumin (BSA; Sigma-Aldrich, St. Louis, MO, USA). The sperm cells were permeabilized using 0.1% Triton X-100 in 0.1% sodium citrate for 2 min in ice and then washed in PBS supplemented with 1% BSA. The positive control was incubated with 100 IU DNAse I (Roche Diagnostic GmbH) for 10 min at 37 °C while the test sample and the negative control were kept on ice. The three pellets were incubated in 45 µL of labelling solution plus 5 µL of terminal deoxynucleotidyl transferase (TdT) enzyme (Roche Diagnostic GmbH) for 1 h at 37 °C in the dark. The negative control was incubated without TdT enzyme. Finally, samples were washed twice using 1% BSA in PBS. The results were analyzed by flow cytometry with a resuspension of pellet in 1 mL of PBS. Green fluorescence (TUNEL-positive cells) was measured using 530 nm ± 30 nm band-pass filter for a total of 10,000 events at 200–300 cells/s on a flow cytometer (FACSCalibur; Becton Dickinson, NJ, USA). Data were processed by CELLQUEST analysis software (Becton Dickinson).

The Comet assay was performed in both alkaline and neutral conditions, according to the methodology previously described by our research group [47]. Samples were washed and adjusted to 10 × 10^6^ sperm/mL. The sperm solution was mixed with low-melting-point agarose, fixed on a slide and stored at 4  °C to jellify. After sperm nucleus decondensation by two consecutive lysis solutions, a denaturing treatment in NaOH solution and electrophoresis at 20 V (1 V/Cm) for 4 min in 0.03 M NaOH buffer was performed for the alkaline Comet slide, and electrophoresis for 12.5 min in TBE buffer and a washing in 0.9% NaCl solution was performed for the neutral Comet slide. Then, the slides were washed in a neutralization solution at neutral pH, dehydrated and dried before stain with DAPI SlowFade Gold anti-fade (Invitrogen, Carlsbad, CA, USA) to classify 400 sperm as fragmented or non-fragmented. The samples were analyzed at CIMAB Barcelona Male Infertility Centre and evaluated by a single researcher under a fluorescence microscope to reduce intra-observer variability. The DNA damage results are given as a percentage of sperm with SDF for each sample (based in the criteria reported before [29]); also as Olive tail moment (OTM) values, obtained with Komet 7 analysis software (Andor, Oxford, UK), thus giving an idea of the DNA damage intensity.

### 2.4. Sperm Chromatin Condensation Analysis

**Figure 1:**
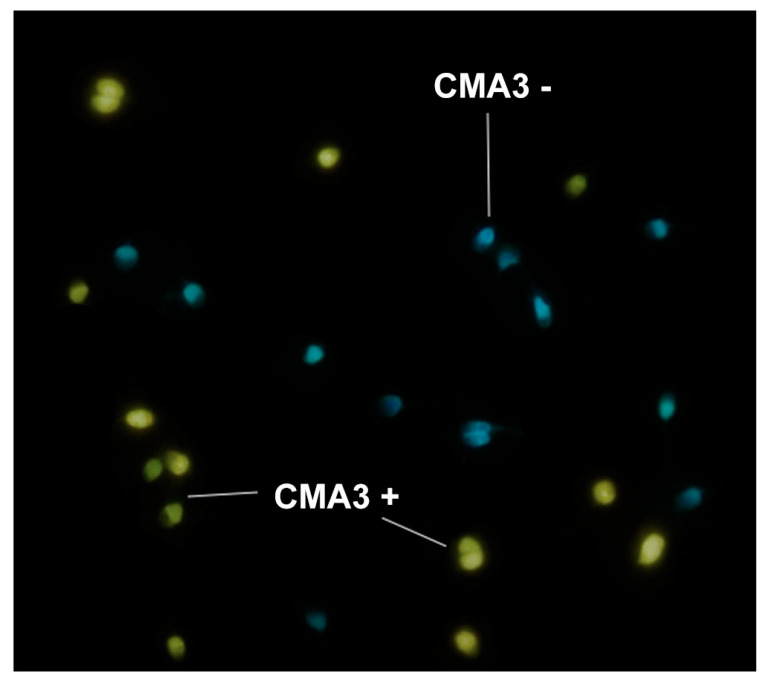
Sperm cells slide stained with Chromomycin A3 and counterstained with DAPI SlowFade Gold antifade. Cells with chromatin condensation deficiency are showed in yellow and normal cells in blue.

A sperm chromatin condensation was performed using Chromomycin A3 (CMA3, Sigma-Aldrich) test. CMA3 is a yellow fluorescence molecule that competes with protamines, labelling chromatin with protamine deficiency. The semen samples were washed twice in PBS, permeabilized with 0.5% Triton X-100, and resuspended up to a sperm concentration of 10^7^ cells/mL. A cellular extension was performed on a slide and air-dried. The staining solution was prepared with 47.5 µL McIlvaine buffer solution (18 mL citric acid 0.1 M and 41 mL Na_2_HPO_4_ 0.2 M) freshly mixed with MgCl_2_ at 10 mM, plus 10 µL of CMA3 (5 mg/mL). The slide was then incubated with 50 µL of staining solution, covered with a coverslip, at room temperature for 20 min in the dark. Then, the coverslip was gently removed and sperm cells were counterstained with DAPI SlowFade Gold antifade (Invitrogen). A total of 400 cells were analyzed under an epifluorescence microscope and evaluated as positive (CMA3 +) or negative (CMA3 −) to obtain the percentage of chromomycin positive cells (Figure 1).

## 3. Results

### 3.1. MiOXSYS Measurements Variability

**Figure 2:**
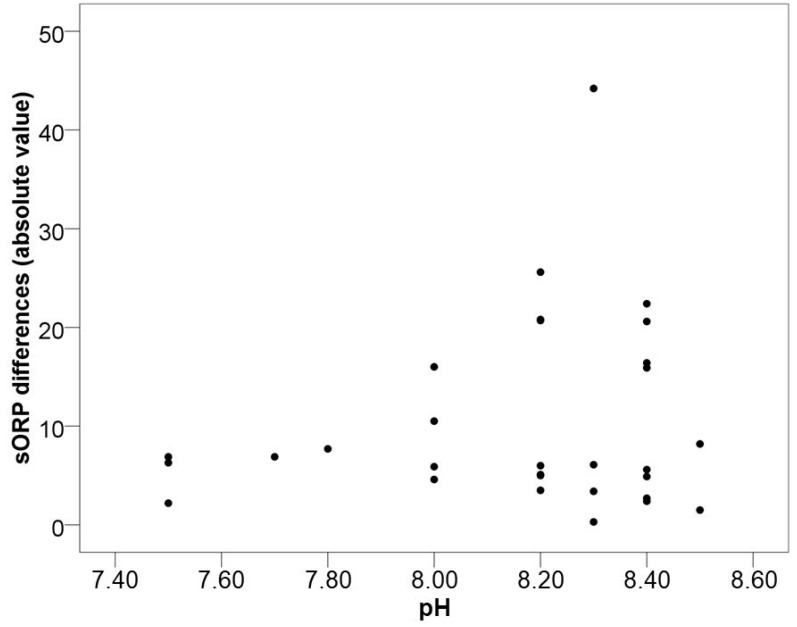
Distribution of differences between sORP1 and sORP2 (absolute values) in idiopathic patients regarding seminal pH values of each sample.

When performing two consecutive measures and studying their behavior, similar ranges and a very strong correlation was observed between them. However, an analysis for paired samples revealed significant differences between both measures (Table 1). The statistical analysis of sORP differences and seminal parameters did not show any statistically significant correlations, but it was observed that samples with a larger difference between the two measures also showed a pH value ≥ 8 (Figure 2).

### 3.2. ORP, DNA Damage and DNA Condensation Relationships

All semen samples were evaluated and the values for the following parameters were recorded: ORP, SDF (TUNEL and Comet assays), chromatin condensation deficiencies, seminal viscosity, seminal pH, sperm concentration, total and progressive motility, normal morphology and seminal volume (Table 2). According to manufacturer instructions, values displayed by the MiOXSYS system need to be normalized by the sperm concentration (nsORP-C) and is sensitive to seminal viscosity. Our results show that the system could also be affected by seminal pH, as seen in the previous section. Thus, to reduce the possible impact of these parameters, two more variables have been generated: sORP normalized by viscosity (nsORP-V) and sORP normalized by pH (nsORP-P) (Table 2).

A comparison of seminal parameters and the three ORP variables are shown in Table 3. Interestingly, all three ORP variables showed a negative correlation to percentage of CMA3 positive cells and to seminal viscosity. nsORP-C and nsORP-P were positively correlated with seminal volume. nsORP-V and nsORP-P displayed a negative correlation with total and progressive motility. nsORP-V was negatively correlated with pH. Finally, no correlation was found between the SDF, sperm morphology and concentration.

The single-strand SDF values assessed by the alkaline Comet assay exhibited a strong negative correlation with total and progressive motility, both for the SDF cell count and software analysis (OTM alkaline) (Table 4). Sperm morphology and concentration seem to follow a trend line, but it was shown to be not significant. The SDF cell count analyzed by TUNEL and alkaline Comet assays were positive correlated with the CMA3 positive cell count (Table 4), except for five patients with low CMA3 positive cells and high single-strand SDF assessed by the alkaline Comet assay. Double-strand SDF assessed by the neutral Comet assay did not correlate with any of the analyzed parameters. The CMA3 positive cell parameter was positively correlated with seminal pH and negatively correlated with progressive motility and seminal volume (Table 4).

## 4. Discussion

Oxidative stress has been pinpointed by several authors as one of the biological processes related to fertility failure [11,13,14,16]. In order to understand the mechanisms by which an individual becomes infertile, it is important to define the relationship among the different infertility biomarkers. Since the oxidative status of a semen sample depends both on ROS and antioxidants, seminal oxidative state assessments must take into account the levels of both agents present in the seminal plasma. In this sense, the ORP values measured by the MiOXSYS system have been proposed as a good biomarker to assess this redox balance [15,48]. Still, the relationship of this system with other seminal parameters is not yet well defined. The aim of this study is to stablish whether a relationship exist between the ORP values and sperm DNA fragmentation and chromatin compaction, and to assess the possible effect of parameters on MiOXSYS system measurements.

### 4.1. MiOXSYS Measurements Variability

Several authors have indicated the influence of seminal viscosity over the MiOXSYS reading outcome as its main disadvantage [23,24]. However, according to the device’s developers, stable, reproducible and reliable results are regularly achieved for each individual, both on fresh and cryopreserved samples [21,49,50]. Despite the good reproducibility described, our results suggest that, beside viscosity, other seminal parameters might be influencing the reading outcome.

After performing two consecutive measurements, results showed that even though there was a correlation between the two measurements, a significant variation was found between them (Table 1). In order to find the cause of this variability, differences between the two sORPs per sample were compared with all the assessed seminal parameters. As shown in Figure 2, the higher the seminal pH, the greater the number of samples with discernible differences between the two MiOXSYS measurements. Interestingly, nsORP-C did not correlate with pH, possibly meaning that sperm count has an important influence on results and that an ejaculate dilution could affect the redox state of the diluted sample.

The MiOXSYS system is an electrochemical method that evaluates the flow of electrons through a galvanostatic sensor. Therefore, it is possible that changes in the number of protons present in the seminal plasma affect the reading outcome. In general, any aqueous solution increases its electrical conductivity the more ions it contains [51,52,53]. Thus, low or high pH values increase electrical conductivity with respect to a neutral pH, possibly altering a sample’s electrical potential measurements. Here we show that the sORP measurement’s reproducibility is affected in samples with alkaline pH values ≥ 8. Starting at pH 8, differences of more than 10 mV between the two sample measurements can be found, reaching up to 44 mV in one case (Figure 2). Further research increasing the number of measurements and samples analyzed should be performed in order to determine the extent of seminal pH influence on the MiOXSYS system reproducibility and the need to include pH value in the normalization algorithm in addition to sperm concentration and viscosity.

### 4.2. Sperm Parameters Relationship

Seminal quality appears to be actively affected by oxidative stress. It apparently plays a central role in the activation or regulation of several mechanisms involved in fertility [13,14,15,16]. Correlations between sORP and semen parameters included in the diagnostic guidelines issued by the WHO in 2010 [43] have already been described. Thus, it has been described that sORP presents a negative correlation with motility, morphology, concentration and volume [45,54,55], meaning that when seminal plasma ORP increases, semen quality decreases. When compared to motility, the three different normalized ORP measures of our study showed correlation coefficients similar to those observed by other authors [45,55]. However, nsORP-C correlations did not achieve statistical significance, probably due to the limited sample size. We did observe a significant positive correlation of nsORP-C and nsORP-P with the seminal volume, a relationship that is explained below. No correlation was found with morphology or sperm concentration (Table 3).

On the other hand, our results show a negative correlation between ORPs and viscosity and also between nsORP-V and pH (Table 3), reinforcing the results described in the previous section regarding measures’ variabilities.

Interestingly, the highest correlation was found when analyzing the relationship between sORP values and the percentage of CMA3 positive cells (Table 4). According to our results, the DNA condensation degree of sperm chromatin is negatively correlated with all three sORP variables, so that the higher the oxidative stress, the fewer cells with altered chromatin compaction. This apparently contradictory result could be explained by the fact that alterations in histone–protamine exchange occur during spermiogenesis in testis. According to the double-step hypothesis proposed by Aitken and De Iuliis [42,56], a defect in DNA condensation would be the first step that would facilitate greater access to ROS, leading to extensive DNA damage. For this reason, a positive correlation is found between the number of sperm with single-strand DNA fragmentation (Alkaline Comet and TUNEL assays) and with compaction alterations assessed by CMA3 (Table 4), in accordance with the previous studies of our group [36] and other authors [38,39]. Then, in epididymis, ROS levels would be balanced by providing antioxidants to generate a suitable conservation medium for sperm. Any alteration in this antioxidant contribution would maintain high levels of ROS all the way through vas deferens, generating even more DNA damage. Finally, to counterbalance this oxidative environment, a lot of seminal liquid would be added to dilute the ROS presence along with large amounts of antioxidants, resulting in a low ORP and high seminal volume. In individuals with basal or slightly raised levels of testicular ROS and without alterations in the histone–protamine exchange, fewer antioxidants would be supplied in the seminal fluid, resulting in a comparatively higher ORP than in the previous case.

The results for DNA integrity suggest no direct correlation between seminal oxidation-reduction potential and fragmentation of sperm DNA, as assessed by either TUNEL or Comet assays (Table 4). Previous studies have shown a direct relationship between ROS levels and single-strand SDF [10,33,34]. The lack of a similar relationship between sORP and SDF suggests that, similarly to what has been commented on the previous paragraph, the incorporation of antioxidants in seminal plasma compensates for testicular ROS levels. High levels of ROS can be offset by high levels of antioxidants that prevent SDF generation, and conversely, basal ROS levels along with a decrease in seminal antioxidants could lead to increased SDF [17,18,19,20]. The SDF generated by ROS occurs mainly in the epididymis and vas deferens but not in the ejaculation stage [57,58,59]. SDF can also be produced by intracellular ROS [60]. Further, there are seemingly contradictory results where some authors observed a positive correlation using a sperm chromatin dispersion test [61,62,63] but another did not find it using a sperm chromatin structure assay [34]. The disparity of techniques used can become biased when comparing results.

Even so, TUNEL and Comet alkaline assays negatively correlate with sperm motility and, in the case of computational analysis of alkaline comet assay, with morphology (Table 4), an association with seminal quality which has been previously described [25,26,27,28,30,33].

According to previous analysis of our group [36], post-ejaculation SDF dynamics is highly variable in both control and infertile patients. Regardless of baseline levels, the generation of new SDF in ejaculate is very different in all studied groups [64,65]. This dynamic could be explained by the interindividual variability observed with sORP, with high sORP individuals generating new SDF over prolonged seminal plasma exposure. In this sense, the analysis of the ORP may be useful in programming the ejaculate management during an assisted reproduction process or research project, such as a rapid isolation of the sperm cells from seminal plasma in high sORP samples to avoid an increase in SDF.

## 5. Conclusions

The tendency observed between sORP1–sORP2 differences and the pH value suggests that, together with seminal viscosity, pH might be affecting the reading outcome of the MiOXSYS system. Further studies will be needed to establish the effect of the seminal pH on the system’s reliability. A negative relationship has been found between the ORP values and the state of sperm chromatin compaction, as well as with seminal quality, but not with SDF. These relationships have allowed us to contribute new information that supports the model proposed by Aitken and De Iuliis on the origin of DNA damage. Finally, our work shows that the combined knowledge of SDF, chromatin compaction and sORP may have relevant clinical applications, particularly when choosing the most appropriate strategy for handling seminal samples in assisted reproduction processes.

## Figures and Tables

**Table 1:** Descriptive statistics of first measure (static oxidation-reduction potential 1; sORP 1) and second measure (sORP 2) displayed by the MiOXSYS system and relationship study between them.

**Measures**	**Mean ± Standard Deviation**	**95% Confidence Interval**
**sORP1 (*n* = 37)**	57.01 ± 27.32	47.90–66.12
sORP2 (*n* = 32) *	55.71 ± 25.84	46.39–65.02
**Statistical test**	**Statistical value**	***p*-value**
**Pearson correlation** **(sORP1 vs sORP2)**	r = 0.853	<0.001
**Paired samples T-student** **(sORP1 vs sORP2)**	t = 2.306	0.028

* Five samples only presented one measurement because the second one was displayed as too low to give a numerical value by the device.

**Table 2:** Distribution of semen parameters in the studied patients.

Semen Parameters	Mean ± Std. Deviation	95% Confidence Interval
**nsORP-C (mV/10^6^ sperm/mL)**	3.02 ± 9.50	−0.35–6.39
**nsORP-V (mV/cps)**	9.84 ± 6.33	7.66–12.02
**nsORP-P (mV/U pH)**	7.05 ± 3.52	5.86–8.24
**TUNEL (%)**	42.23 ± 14.98	37.37–47.09
**Alkaline comet (%)**	34.33 ± 15.07	29.64–39.03
**OTM alkaline comet**	0.82 ± 0.23	0.75–0.90
**Neutral comet (%)**	66.17 ± 15.63	61.3–71.04
**OTM neutral comet**	0.62 ± 0.16	0.57–0.68
**CMA3 (%)**	38.06 ± 16.51	32.78–43.34
**Viscosity (cps)**	9.13 ± 13.34	4.80–13.45
**pH**	8.13 ± 0.39	8.01–8.26
**Sperm concentration (10^6^ sperm/mL)**	68.14 ± 57.49	48.97–87.30
**Total motility (%)**	58.85 ± 16.50	53.35–64.35
**Progressive motility (%)**	46.69 ± 19.03	40.35–53.04
**Normal morphology (%)**	11.36 ± 12.74	6.42–16.30
**Seminal volume (mL)**	3.00 ± 1.46	2.53–3.46

nsORP-C: ORP normalized by sperm concentration; nsORP-V: ORP normalized by seminal viscosity; nsORP-P: ORP normalized by pH; CMA3: Chromomycin A3 test; OTM: Olive tail moment.

**Table 3:** sORP correlation analysis with semen and sperm parameters with Pearson’s (r) and Spearman’s (ρ) correlation test as it matches. Confirmation with an inverse curvilinear estimation (r2) in the required case.

Semen Parameters	nsORP-C	nsORP-V	nsORP-P
**TUNEL (%)**	ρ = 0.160	ρ = 0.173	ρ = 0.213
**Alkaline comet (%)**	ρ = 0.125	r = −0.005	r = 0.061
**OTM alkaline comet**	ρ = −0.039	r = −0.252	r = 0.125
**Neutral comet (%)**	ρ = −0.249	r = 0.036	r = −0.054
**OTM neutral comet**	ρ = 0.129	ρ = 0.338	ρ = −0.028
**CMA3 (%)**	ρ = −0.394 *	ρ = −0.545 **	ρ = −0.337*
**Viscosity (cps)**	ρ = −0.009 r^2^ = 0.138 *	—	ρ = −0.416 *
**pH**	ρ = −0.002	ρ = −0.347 *	—
**Sperm concentration (10^6^ sperm/mL)**	—	ρ = 0.089	ρ = 0.081
**Total motility (%)**	ρ = −0.198	r = −0.477 **	r = −0.355 *
**Progressive motility (%)**	ρ = 0.125	r = −0.485 **	r = −0.445 *
**Normal morphology (%)**	ρ = −0.138	ρ = −0.005	ρ = 0.163
**Seminal volume (mL)**	ρ = 0.452 **	r = −0.058	r = 0.342 *

* *p*-value < 0.05; ** *p*-value < 0.01.

**Table 4:** Sperm DNA fragmentation (SDF) and chromomycin a3 (CMA3) correlation analysis with semen and sperm parameters with Pearson’s and Spearman’s correlation test as it matches.

Semen Parameters	TUNEL	Alkaline Comet	OTM Alkaline	Neutral Comet	OTM Neutral	CMA3
**CMA3 (%)**	0.338 *	0.346 *	−0.043	−0.043	−0.230	—
**Viscosity (cps)**	−0.125	0.321 *	0.390 *	−0.083	−0.463 **	0.202
**pH**	0.166	−0.165	−0.229	0.004	−0.018	0.395 **
**Sperm concentration (10^6^ sperm/mL)**	−0.048	−0.214	−0.106	0.210	−0.197	0.242
**Total motility (%)**	−0.037	−0.363 *	−0.372 *	0.022	−0.016	−0.024
**Progressive motility (%)**	−0.384 *	−0.417 **	−0.349 *	−0.059	−0.175	−0.391 *
**Normal morphology (%)**	0.010	0.080	0.215	−0.161	−0.228	0.099
**Seminal volume (mL)**	−0.006	−0.147	0.108	−0.300	−0.230	−0.363*

OTM: Olive tail moment; CMA3: Cromomycin A3 test. * *p*-value < 0.05; ** *p*-value < 0.01.
